# Supplementary material for: Probing Limits of Information Spread with Sequential Seeding
Source: Sci Rep. 2018 Sep 18;8:13996. doi: 10.1038/s41598-018-32081-2 (PMC6143613; doi:10.1038/s41598-018-32081-2)
Supplement: Supplementary file 1 — Supplementary information [file 41598_2018_32081_MOESM1_ESM.pdf]

Probing Limits of Information Spread with Sequential Seeding

Jarosław Jankowski[1,*], Bolesław K. Szymański[2], Przemysław Kazienko[3], Radosław Michalski[3], and Piotr Bródka[3]

[1]Faculty of Computer Science and Information Technology, West Pomeranian University of Technology, 70-310 Szczecin, Poland
[2]Social and Cognitive Networks Academic Research Center and Department of Computer Science, Rensselaer Polytechnic Institute, Troy NY 12180, USA
[3]Faculty of Computer Science and Management, Wroclaw University of Science and Technology, 50-370 Wroclaw, Poland
[*]jjankowski@wi.zut.edu.pl

ABSTRACT

This is the supplementary information related to the manuscript *„Probing Limits of Information Spread with Sequential Seeding"*.

Supplementary Information

Experimental results for undirected networks

Each from 162 combinations from configuration space $N \times PP \times SP \times R$ was applied as a simulation configuration to single stage SN and sequential seeding SQ with the use of coordinated execution. Repeated simulations resulted in 16,200,000 simulation cases. Results from individual simulation cases are presented in Fig. 1 (A). They show that C_{SQ} performs better than C_{SN} for most cases with coverage $C_{SQ} \in [30\%, 85\%]$.

Fig. 1 (B) shows coverage for sequential approaches C_{SQ} compared to single stage methods C_{SN}. The maximum coverage C_{Max} for optimal seed set is presented as well as the upper bound $C_{GreedySN} * e/(e-1)$ for all cases. The results show that sequential seeding based on simple degree-based ranking in 92.2% of cases outperforms single stage computationally expensive greedy heuristic, see Fig. 1 (B1). Moreover, sequential seeding based on degree $C_{degreeSQ}$ is greater than sequential greedy approach $C_{greedySQ}$ in 62.6% of cases.

For all simulation cases C_{SQ} shows better results than C_{SN} in 96.7% of cases what as presented in Fig. 1 (C). Increase above 5% was achieved in 20.2% of simulation cases. The obtained coverage was dependent on seed selection strategy with 96.0% better results for random selection, 100% for degree based selection and 93.9% for greedy based selection, see Fig. 1 (C1).

Fig. 1 (D) shows one of real diffusion cases with visible differences between sequential (SQ) and single stage seeding (SN). As a result of better usage of natural diffusion processes sequential approach outperforms the single stage approach.

Apart from user rankings the results were dependent on the network characteristics with visible differences seen in Fig. 1 (E). Network N6 delivered the highest gain (83%) while the least gain (30%) was achieved for network N3.

Experimental results for directed network

Performance of sequential seeding within directed network was analyzed for random node selection, degree based selection and greedy approach. Detailed results for all used probabilities and strategies for single stage and sequential seeding are presented in Table 1. The highest increase of coverage was observed for sequential degree based selection with results better than greedy selection for both single and sequential seeding. The highest gain for degree based selection was observed for low propagation probabilities 0.05, 0.01, 0.15. For higher probabilities differences between strategies are smaller.

References

1. Newman, M. E. Scientific collaboration networks. i. network construction and fundamental results. *Phys. review E* **64**, 016131 (2001).
2. Opsahl, T. & Panzarasa, P. Clustering in weighted networks. *Soc. networks* **31**, 155–163 (2009).
3. Newman, M. E. The structure of scientific collaboration networks. *Proc. national academy sciences* **98**, 404–409 (2001).

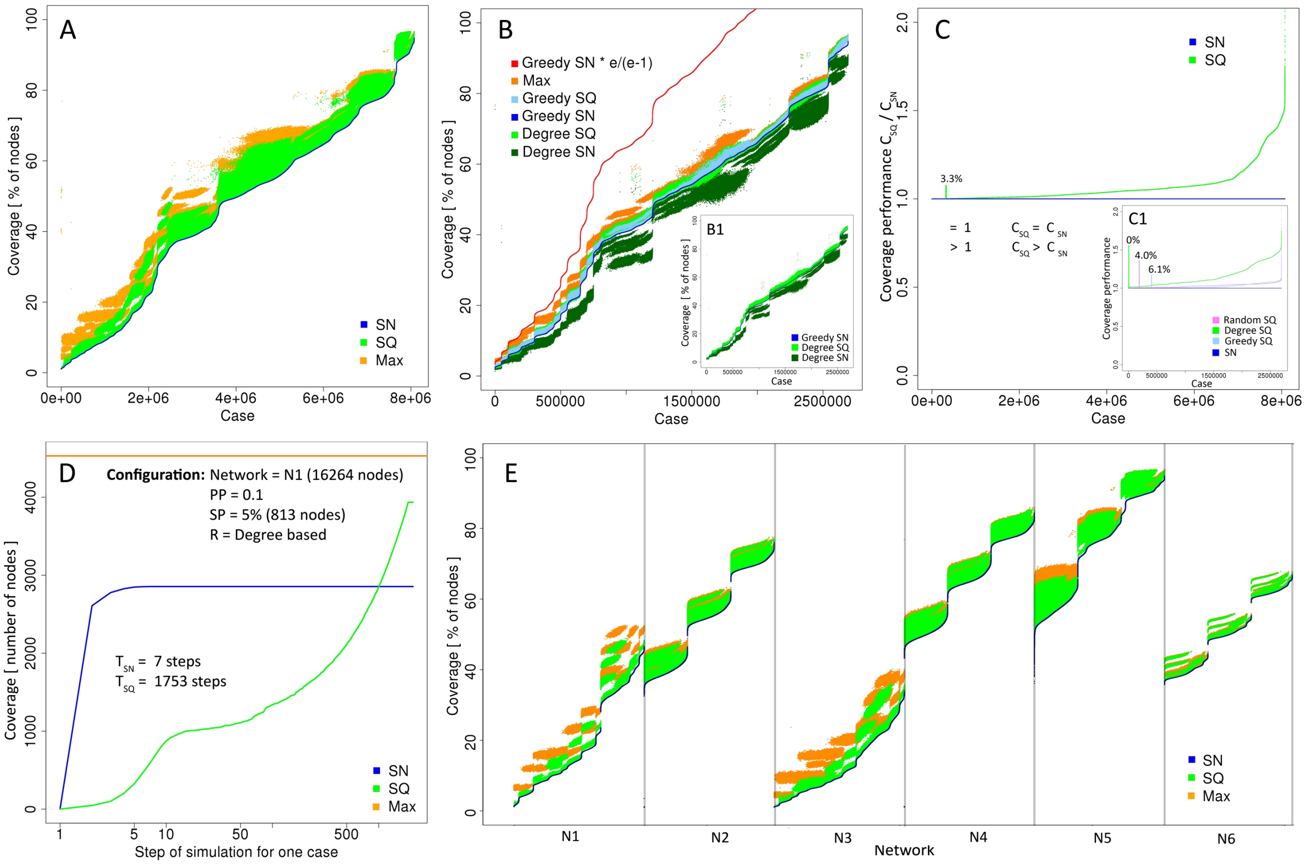

Figure 1. **(A)** Coverage for sequential approach C_{SQ} compared with single stage C_{SN} and maximum coverage C_{Max} for all configurations and all simulation cases; ordered by C_{SN}; **(B)** Coverage as percentage of all activated network nodes for greedy and degree-based single stage and sequential strategies and their relation to C_{Max} and the upper bound; ordered by C_{SN}; **(B1)** Performance of sequential and single stage approaches with degree-based selection in relation to single stage greedy ranking;**(C)** Performance of sequential seeding SQ in the relation to single stage seeding SN, a vertical tick denotes percentage of cases with no gain: $C_{SQ} = C_{SN}$; **(C1)** Performance of random, degree and greedy based sequential seeding compared with single stage seeding SN; **(D)** Steps of one sequential and one single stage diffusion process in the coordinated execution for one configuration; **(E)** Coverage values for sequential C_{SQ} and single stage seeding C_{SN} as well as maximum coverage C_{Max} for networks N1-N6 and all simulation cases.

4. Adamic, L. A. & Glance, N. The political blogosphere and the 2004 us election: divided they blog. In *Proceedings of the 3rd international workshop on Link discovery*, 36–43 (ACM, 2005).

5. Leskovec, J. & Mcauley, J. J. Learning to discover social circles in ego networks. In *Advances in neural information processing systems*, 539–547 (2012).

6. Leskovec, J., Huttenlocher, D. & Kleinberg, J. Predicting positive and negative links in online social networks. In *Proceedings of the 19th international conference on World wide web*, 641–650 (ACM, 2010).

7. Read, K. E. Cultures of the central highlands, new guinea. *Southwest. J. Anthropol.* **10**, 1–43 (1954).

Coverage for random seed selection					
**PP**	**Single stage**	**% of Max**	**Sequential**	**Increase**	**Gain**
0.05	5.43	62.2%	5.67	1.04	7.4%
0.1	7.43	61.5%	8.17	1.09	16.0%
0.15	9.65	67.4%	11.03	1.14	29.5%
0.2	11.76	75.9%	13.56	1.15	48.3%
0.25	13.34	83.9%	15.07	1.13	68.0%
Coverage for degree based seed selection					
**PP**	**Single stage**	**% of Max**	**Sequential**	**Increase**	**Gain**
0.05	5.59	64.0%	5.99	1.07	12.5%
0.1	7.54	62.4%	8.60	1.15	23.4%
0.15	9.56	66.7%	11.39	1.21	38.5%
0.2	11.45	73.9%	13.75	1.22	57.0%
0.25	12.96	81.5%	15.14	1.19	74.5%
Coverage for greedy seed selection					
**PP**	**Single stage**	**% of Max**	**Sequential**	**Increase**	**Gain**
0.05	5.73	65.6%	5.88	1.02	5.0%
0.1	7.88	65.2%	8.47	1.08	14.2%
0.15	10.14	70.8%	11.08	1.09	22.4%
0.2	12.22	78.9%	13.59	1.12	42.0%
0.25	13.72	86.3%	15.09	1.11	62.9%

Table 1. Coverage for single stage and sequential seeding for used seed selection methods and propagation probabilities

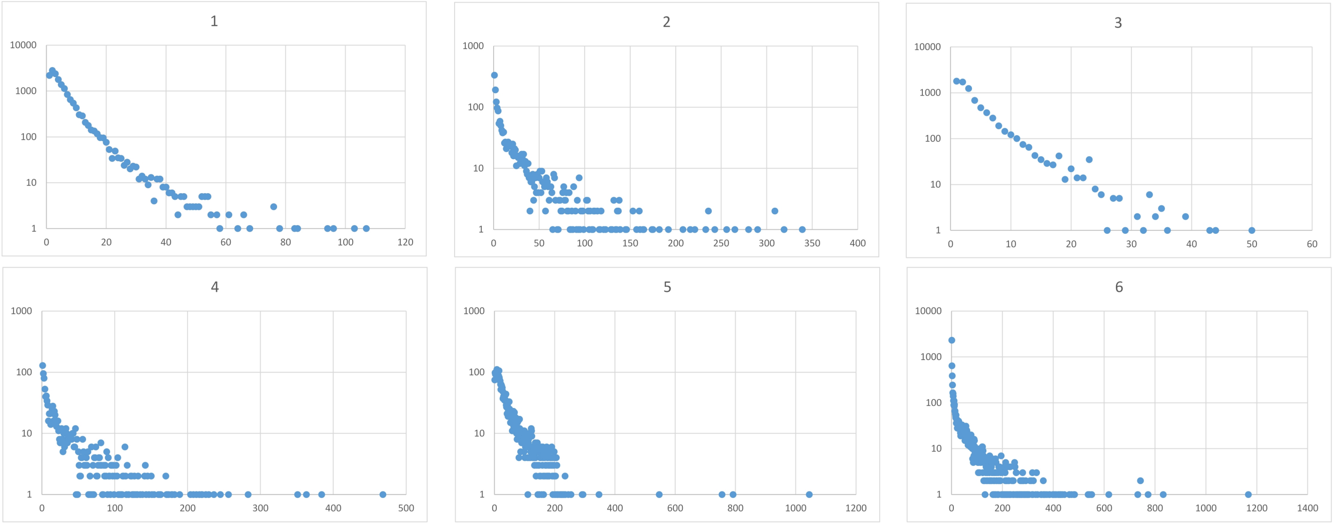

Figure 2. Degree distribution for networks N1-N6

No.	Network name	Ref	Nodes	Edges	Edge type	Components	CC	Diameter
1	N1 - Condensed Matter collaboration	1	16,264	47,594	Undirected	726	0.638	18
2	N2 - Communication network at University of California	2	1,899	20,296	Undirected	4	0.109	8
3	N3 - High-Energy Theory collaboration network	3	7,610	15,751	Undirected	581	0.486	19
4	N4 - Political blogs	4	1,224	19,090	Undirected	2	0.320	8
5	N5 - ego-Facebook	5	4,039	88,234	Undirected	1	0.606	8
6	N6 - wiki-Vote	6	7,115	103,689	Undirected	24	0.141	7
7	N7 - Social network of tribes	7	16	114	Directed	1	0.519	3

Table 2. Description of networks used in the experiments
